# Supplementary material for: EphB2 receptor cell-autonomous forward signaling mediates auditory memory recall and learning-driven spinogenesis
Source: Commun Biol. 2019 Oct 11;2:372. doi: 10.1038/s42003-019-0625-x (PMC6789002; doi:10.1038/s42003-019-0625-x)
Supplement: Supplementary file 1 — Supplementary Information [file 42003_2019_625_MOESM1_ESM.pdf]

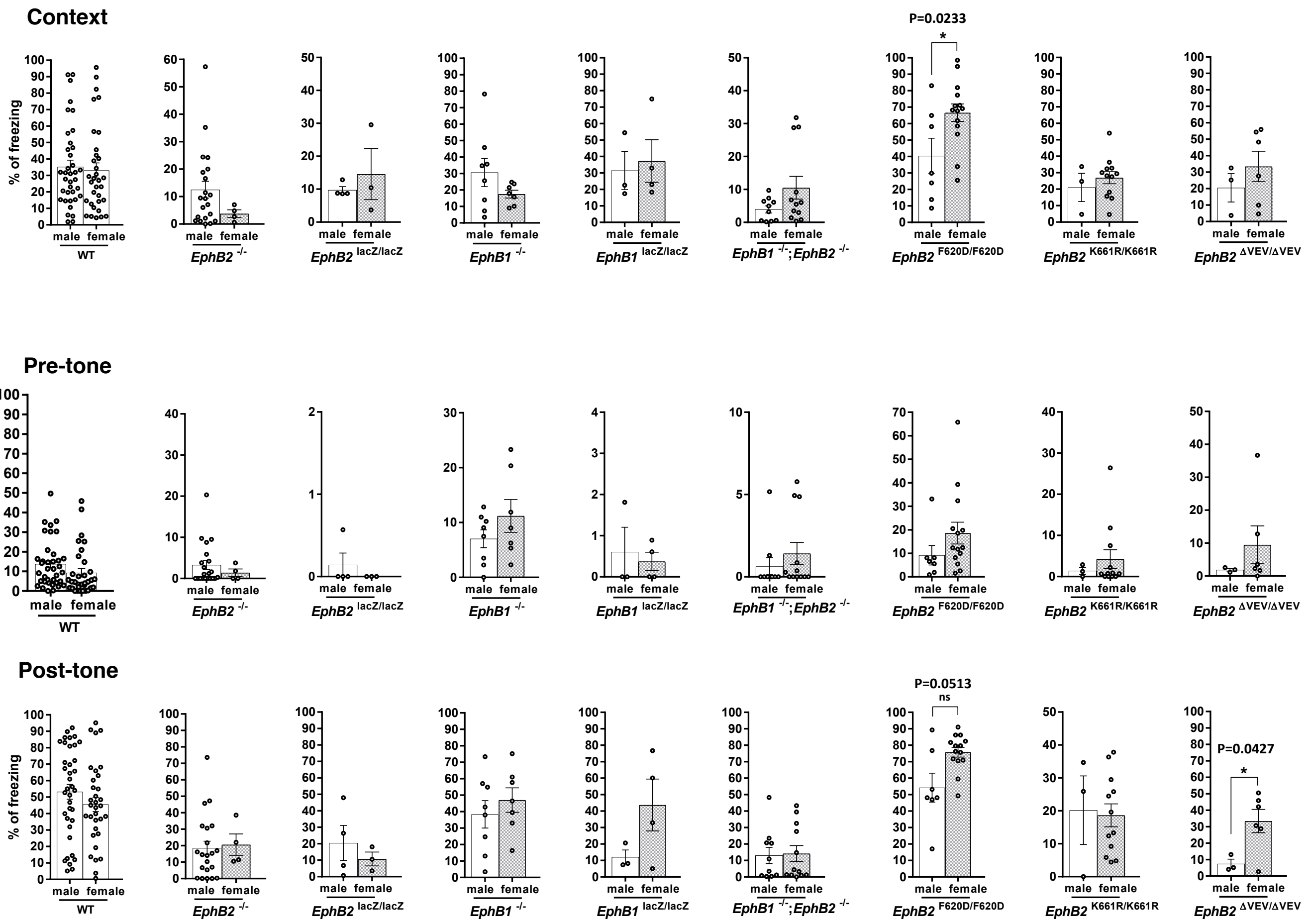

Supplementary Figure 1: Results of fear conditioning freezing data in males and females from WT and the various *EphB2* mutants. The percentage of time freezing was plotted to isolate male and female data for both the context test on day 2 and the pre-tone and post-tone results of the sound-cued test on day 4. n=37M (male)/ 33F(female) in WT, n=21M/4F in *EphB2*$^{-/-}$ knockouts, n=4M/3F in *EphB2*$^{lacZ/lacZ}$ intracellular-truncated, n=8M/7F in *EphB1*$^{-/-}$ knockouts, n=3M/4F in *EphB1*$^{lacZ/lacZ}$ intracellular-truncated, n=10M/12F in *EphB1*$^{-/-}$;*EphB2*$^{-/-}$ double knockouts, n=7M/14F in *EphB2*$^{F620D/F620D}$ kinase-overactive, n=3M/ 12F in *EphB2*$^{K661R/K661R}$ kinase-dead, and n=3M/6F in *EphB2*$^{\Delta VEV/\Delta VEV}$ PDZ domain binding-dead. Two-tailed unpaired t-test were assessed for all groups with *p* values reported/shown here only for relevant cases. Contextual test in *EphB2*$^{F620D/F620D}$, 40.38% ±10.73, n=7 in male and 66.63% ± 5.35, n=14 in female, $t_{(19)}$=2.47, *p*=0.0233; post-tone sound-cued test in *EphB2*$^{F620D/F620D}$, 54.22% ±8.8, n=7 in male and 75.78% ± 2.96, n=14 in female, Welch-corrected $t_{(7.39)}$=2.32, *p*=0.0513; post-tone sound-cued test in *EphB2*$^{\Delta VEV/\Delta VEV}$, 7.48% ±2.83, n=3 in male and 33.46% ± 7.06, n=6 in female; $t_{(7)}$=1.14, *p*=0.0427.

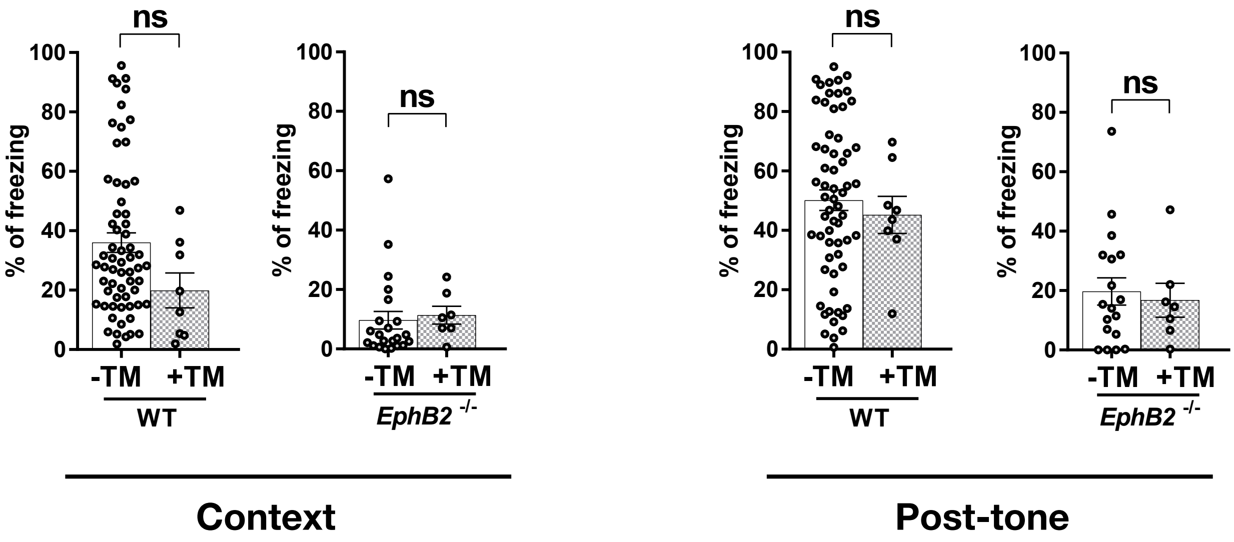

Supplementary Figure 2: No significant effect of tamoxifen on freezing results induced by fear conditioning in WT and *EphB2*$^{-/-}$ mutant mice. The percentage of time freezing was determined from WT or *EphB2*$^{-/-}$ mutant mice subjected to FC either with (+TM) or without (-TM) a single injection of tamoxifen on day 0 immediately prior to the training/encoding session. No significant effect of tamoxifen was observed in the context test on day 2 or the sound-cued test on day 4 using two-tailed unpaired t-tests. Contextual test in WT, 36.01% ± 3.27, n=62 in the absence of tamoxifen and 19.91% ± 5.9, n=8 in presence of tamoxifen, $t_{(68)}=1.71$, $p=0.0912$; contextual test in *EphB2*$^{-/-}$, 9.67% ± 2.98, n=22 in the absence of tamoxifen and 11.39% ± 2.99, n=7 in the presence of tamoxifen, $t_{(27)}=0.3082$, $p=0.7603$; sound-cued test in WT, 50.16% ± 3.47, n=62 in the absence of tamoxifen and 45.24% ± 6.26, n=8 in presence of tamoxifen, $t_{(48)}=0.49$, $p=0.6224$; sound-cued test in *EphB2*$^{-/-}$, 19.72% ± 4.6, n=18 in the absence of tamoxifen and 16.77% ± 5.71, n=7 in the presence of tamoxifen, $t_{(23)}=0.36$, $p=0.7240$.
