# Supplementary material for: EphB2 receptor cell-autonomous forward signaling mediates auditory memory recall and learning-driven spinogenesis
Source: Commun Biol. 2019 Oct 11;2:372. doi: 10.1038/s42003-019-0625-x (PMC6789002; doi:10.1038/s42003-019-0625-x)
Supplement: Supplementary file 2 — Description of Additional Supplementary Files [file 42003_2019_625_MOESM2_ESM.docx]

**File:** Supplementary Data 1.xlsx

**Description:** Source Data for figures 1-8.
